# Structural and Proteomic Studies of the *Aureococcus anophagefferens* Virus Demonstrate a Global Distribution of Virus-Encoded Carbohydrate Processing

**DOI:** 10.3389/fmicb.2020.02047

**Published:** 2020-09-08

**Authors:** Eric R. Gann, Yuejiao Xian, Paul E. Abraham, Robert L. Hettich, Todd B. Reynolds, Chuan Xiao, Steven W. Wilhelm

**Affiliations:** ^1^Department of Microbiology, The University of Tennessee, Knoxville, Knoxville, TN, United States; ^2^Department of Chemistry and Biochemistry, The University of Texas at El Paso, El Paso, TX, United States; ^3^Chemical Sciences Division, Oak Ridge National Laboratory, Oak Ridge, TN, United States

**Keywords:** giant viruses, viro-cell metabolism, proteomics, cryo-EM, carbohydrate lyases

## Abstract

Viruses modulate the function(s) of environmentally relevant microbial populations, yet considerations of the metabolic capabilities of individual virus particles themselves are rare. We used shotgun proteomics to quantitatively identify 43 virus-encoded proteins packaged within purified *Aureococcus anophagefferens* Virus (AaV) particles, normalizing data to the per-virion level using a 9.5-Å-resolution molecular reconstruction of the 1900-Å (AaV) particle that we generated with cryogenic electron microscopy. This packaged proteome was used to determine similarities and differences between members of different giant virus families. We noted that proteins involved in sugar degradation and binding (e.g., carbohydrate lyases) were unique to AaV among characterized giant viruses. To determine the extent to which this virally encoded metabolic capability was ecologically relevant, we examined the TARA Oceans dataset and identified genes and transcripts of viral origin. Our analyses demonstrated that putative giant virus carbohydrate lyases represented up to 17% of the marine pool for this function. In total, our observations suggest that the AaV particle has potential prepackaged metabolic capabilities and that these may be found in other giant viruses that are widespread and abundant in global oceans.

## Introduction

The most biologically complex virus particles may be the “giants” that belong to the Nucleocytoplasmic Large dsDNA Virus (NCLDV) group. These viruses have genomes and particle sizes that can rival bacteria in size (Wilhelm et al., 2017). Moreover, they have been isolated from numerous environments (Legendre et al., 2015; Abrahao et al., 2018), are abundant in the world’s ocean (Moniruzzaman et al., 2020), and have been shown to be important in constraining productivity in many distinct ecosystems (Vardi et al., 2012), suggesting that their life strategy is ubiquitous and effective. The genetic repertoire associated with these large genomes allows for enhanced independence from the host’s transcriptional (Mutsafi et al., 2010) and translational (Abrahao et al., 2018) machinery during infection. Importantly, the size and complexity of the virion particle allow for the packaging of not only structural proteins but also enzymes (Dunigan et al., 2012; Fischer et al., 2014) important in the initial stages of the infection (Zhang et al., 2011; Milrot et al., 2017) that, in effect, give these particles biochemical potential.

Nucleocytoplasmic Large dsDNA Virus members play important roles in algal bloom collapse (Gastrich et al., 2004; Vardi et al., 2012). The eukaryotic alga *Aureococcus anophagefferens* causes recurrent algal blooms off the eastern coast of the United States, negatively impacting the economy by disrupting tourism and devastating fisheries (Gobler and Sunda, 2012). Large, icosahedral viruses can infect natural *A. anophagefferens* populations, with an increased prevalence of infected cells at bloom collapse (Gastrich et al., 2004). The lab model for this system, the *A. anophagefferens* Virus (AaV), belongs to the extended-Mimiviridae family and has a 371-kbp genome that encodes 377 coding sequences and 8 tRNAs (Moniruzzaman et al., 2014). The suite of proteins encoded by these virus particles have been described as being more consistent with “cellular” life forms than viruses (Wilhelm et al., 2016). Previous transcriptomic analyses performed over the course of the infection cycle show that transcripts for all but two of these coding sequences were detected over the 21-h infection cycle (Moniruzzaman et al., 2018). However, little is known about the initial, critical moments of the infection cycle and the roles of viral vs. host proteins. Indeed, given the abundance of these viruses in nature yet paucity of NCLDV model systems and information on their physiological ecology, complete characterization of lab models to infer environmental function is a necessity. This is particularly true for the virus particle and its potential to have biochemical activity at the onset of the interaction with the host, as well as once released into the environment.

Here, we focused on the AaV particle, by first reconstructing its capsid structure using cryogenic electron microscopy (Cryo-EM). A knowledge of particle structure and the components needed to assemble it allowed us to quantify the proteins packaged in purified particles (determined by proteomics) at the per virion level. We used this opportunity to compare both the structure and protein ensemble to other giant virus particles to further understand similarities and differences that have occurred since evolution from a common ancestor. Because several proteins packaged were involved in sugar metabolism, we also determined the distribution of one type, putative polysaccharide lyases, to approximate virus-encoded metabolic capabilities (i.e., polysaccharide degrading) in the world’s oceans. This work provides further evidence of the importance of viruses in shaping the biogeochemical potential of not only infected host cells, but potentially the community as a whole in marine systems.

## Materials and Methods

### Study Design

The goal of this research was to determine the protein complement of the AaV particle and whether there was a conservation of packaged proteins among giant viruses. To do this, we first grew and infected cultures in the lab to produce quantities of AaV for purification and structural determination by Cryo-EM. Structural determination allowed us to predict the number of major capsid proteins (MCP) per virus particle, and then we used this abundance to normalize proteomic data to individual proteins per particle. Cryo-EM and proteomics were accomplished using standard approaches: with replication: for LC/MS/MS (true triplicate biological samples, with viruses produced from different host batches at ∼ 2-month intervals) and for Cryo-EM (batches of lysate were produced over 4 years). We bioinformatically compared proteomes of individual particles across the characterized giant viruses to determine whether there were structural or functional trends of the particles themselves. After identifying protein contents of the individual virus particles and the observation of novel sugar-degrading capabilities of virus particles, we chose the TARA Oceans dataset as it contained the most complete global metagenomic and metatranscriptomic that were available.

### AaV Propagation and Concentration

Non-axenic *A. anophagefferens* CCMP1984 was grown in a modified ASP_12_A (Gann, 2016), at 19°C with a 14:10 light–dark cycle that included an irradiance level of 90 μmol photons m^–2^ s^–1^. Cultures were infected with fresh AaV lysate from which cellular debris was removed by a 0.45-μm pore-size syringe filter (Millex-HV 0.45-μm nominal pore-size PVDF, Millipore Sigma, United States). Lysates were pooled (>80 L) and concentrated by tangential flow filtration (Fischer et al., 2014). First, lysate was concentrated 50× using a Proflux M12 Tangential Flow Filtration System (MilliporeSigma, United States) equipped with a 30-kDa Helicon S10 Ultrafiltration Spiral Cartridge Filter (MilliporeSigma, United States) and then further concentrated using a Lab-scale Tangential Flow Filtration System (Fisher Scientific, United States) equipped with a Durapore 30-kDa Pellicon XL Filter (MilliporeSigma, United States). Bacteria were removed by pelleting 50-ml concentrates in serial centrifugations (5,000 × *g*, 5 min) in a Sorvall Lynx 4000 Centrifuge (Thermo Fisher Scientific, United States) with a Fiberlite F14-14 × 50cy rotor (Thermo Fisher Scientific, United States) and then by filtration using a 0.45-μm pore-size syringe filter (Millex-HV 0.45 μm nominal pore-size PVDF, Millipore Sigma, United States). Samples for electron microscopy were stored at 4°C in a final concentration of 1% Triton X-100, while those for proteomics were stored at −20°C without detergent.

### Electron Microscopy

Purified AaV samples were divided into 1-ml aliquots in 1.5-ml microcentrifuge tubes and centrifuged at 1,000 × *g* for 1 h. To remove the detergent leftover from previous steps, AaV pellets were collected and slowly diluted 200× with Tris-Base buffer A (50 mM Tris, 500 mM NaCl, and 5 mM MgCl_2_, pH 8.0) using a Fusion 200 syringe pump (Chemyx, United States) at a flow rate of 0.02 ml/min with the sample tube shaking. Next, the diluted AaV sample was concentrated by centrifugation at 1,000 × *g* for 1 h in 1-ml aliquots and again slowly diluted with Tris-Base buffer B (50 mM Tris and 5 mM MgCl_2_, pH 8.0) to reach a final salt concentration of 250 mM. Finally, the AaV sample was concentrated to a volume less than 10 μl by centrifugation. Cryo-EM specimens were prepared by adding 3.5 μl of the final AaV sample on a Quantifoil R 3.5/1 grids (Quantifoil Micro Tools GmbH, Germany), blotting manually, and vitrified using a guillotine style plunging device into liquid ethane at liquid nitrogen temperature. Cryo-EM data were collected at the NIH-funded regional cryo-EM consortia at UCLA and Stanford SLAC on Titan Krios G3 microscopes (Thermo Fischer Scientific, United States) equipped with Gatan BioQuantum Energy filter and K2 detector in EFTEM mode with nanoprobe. The reported magnification was 105,000×, resulting in pixel sizes of 1.36 and 1.34 Å/pixel in the collected movies from UCLA and Stanford SLAC, respectively. Movies were collected for 7 s at 5 frames/s. The total dose level was at approximately 30 e-/Å^2^. The movies were motion corrected and dose weighted using MotionCor2 (Zheng et al., 2017) to generate final micrographs. A total of 570 particles were boxed from the micrographs using e2boxer.py (Tang et al., 2007) and used for particle reconstruction by the program Relion3 (Zivanov et al., 2018). As the primary focus of this study was the viral capsid formed by MCPs, a soft mask was applied in the 3D refinement to remove the inner membrane and the viral genome sack, which resulted in significant improvement in the resolution of the viral capsid. The resolution of the reconstruction was 9.5 Å determined by the gold standard Fourier Shell correlation (FSC) with a threshold of 0.143. Electron microscopy data were deposited to the Electron Microscopy Data Bank under the access code: EMD-22339.

### Protein Extraction and Digestion

Concentrated lysates (4 ml) were further concentrated using 10-kDa molecular weight spin columns (Vivaspin 2, GE Health, United States) and resuspended in sodium deoxycholate (SDC) buffer (2% in 100 mM of NH_4_HCO_3_, 10 mM dithiothreitol). Cysteines were blocked by adjusting each sample to 30 mM iodoacetamide and incubated in the dark for 15 min at room temperature. Proteins were digested *via* two aliquots of sequencing-grade trypsin [1:75 (w:w), Promega, United States], first overnight followed by a 3-h incubation at 37°C. The peptide flow through was collected *via* centrifugation and the sample was adjusted to 1% formic acid to precipitate SDC. Hydrated ethyl acetate was added to each sample at a 1:1 (v:v) ratio three times to effectively remove SDC. Samples were then placed in a SpeedVac Concentrator (Thermo Fischer Scientific, United States) to remove ethyl acetate and further concentrate the sample. The peptide-enriched flow through was quantified by BCA assay (Pierce Biotechnology, United States), desalted on RP-C18 stage tips (Pierce Biotechnology, United States), and then stored at −80°C.

### Protein Identification and Quantification

Peptide mixtures were analyzed using an established high-performance peptide sequencing approach (Villalobos Solis et al., 2019) on a Q-Exactive Plus mass spectrometer (Thermo Fischer Scientific, United States) coupled with a Proxeon EASY-nLC 1200 liquid chromatography (LC) pump (Thermo Fisher Scientific, United States). In brief, peptides were separated on a 75-μm-inner-diameter microcapillary column packed with 40 cm of Kinetex C18 resin (1.7 μm, 100 Å, Phenomenex). For each sample, a 2-μg aliquot (determined by BCA assay—above) was loaded in buffer A (0.1% formic acid, 2% acetonitrile) for 10 min, eluted with a linear 90 min gradient of 2–20% of buffer B (0.1% formic acid, 80% acetonitrile), followed by an increase in buffer B to 30% for 10 min, another increase to 50% buffer for 10 min, concluding with a 10-min wash at 98% buffer A. The flow rate was kept at 200 nl min^–1^. MS data were acquired with the Thermo Xcalibur software version 4.27.19 (Thermo Fischer Scientific, United States) using the data-depending acquisition top10 method where up to 10 different precursor *m*/*z* values can be triggered for fragmentation per full scan. Target values for the full scan MS spectra were 1 × 10^6^ charges in the 300–1,500 m/z range with a maximum injection time of 25 ms. Transient times corresponding to a resolution of 70,000 at m/z 200 were chosen. A 1.6 m/z isolation window and fragmentation of precursor ions was performed by higher-energy C-trap dissociation (HCD) with a normalized collision energy of 27. MS/MS scans were performed at a resolution of 17,500 at m/z 200 with an ion target value of 1 × 10^–5^ and a maximum injection time of 50 ms. Dynamic exclusion was set to 20 s to avoid repeated sequencing of peptides.

The MS raw data files were processed using the commercial software Proteome Discoverer v2.2 (Thermo Fischer Scientific, United States). Raw data files were searched against both the *A. anophagefferens* (Gobler et al., 2011) and AaV (Moniruzzaman et al., 2014) reference proteome to which common contaminate proteins had been added. A decoy database, consisting of the reversed sequences of the target database, was appended to discern the false-discovery rate (FDR) at the spectral level. For standard database searching, the peptide fragmentation spectra (MS/MS) were analyzed by Proteome Discoverer v2.2 (Thermo Fischer Scientific, United States). The MS/MS was searched using the MS Amanda v2.0 (Dorfer et al., 2014) and was configured to derive fully tryptic peptides using settings for high-high MS/MS data: MS1 mass tolerance of 10 ppm and MS2 mass tolerance of 0.02 Da. A static modification on cysteines (iodoacetamide; +57.0214 Da) and a dynamic modification on methionine (oxidation; 15.9949) were considered. The results were processed by Percolator (The et al., 2016) to estimate *q*-values. Peptide spectrum matches (PSMs) and peptides were considered identified at a *q*-value < 0.01. For label-free quantification, MS1-level precursor intensities (area) were derived from the Minora Feature and Precursor ions quantifier nodes using default parameters. Missing values were imputed (Low abundance resampling method) and proteins were normalized by the total peptide amount using Proteome Discoverer (Thermo Fischer Scientific, United States).

### Bioinformatic Comparison of Packaged Proteins Within Different Viral Families

To compare the protein complement of AaV with other NCLDV members, viral protein sequences and locations of coding regions within the genomes from NCBI were downloaded (Brister et al., 2015). Viruses with particle proteomes used in this study included *Acanthamoeba polyphaga* Mimivirus (APMV) (Renesto et al., 2006), *Cafeteria roenbergensis* Virus (CroV) (Fischer et al., 2014), *Emiliania huxleyi* Virus 86 (EhV) (Allen et al., 2008), *Marseillevirus* (Boyer et al., 2009), *Melbournevirus* (Okamoto et al., 2018), *Pithovirus sibericum* (Legendre et al., 2014), *Mollivirus sibericum* (Legendre et al., 2015), *Pandoravirus dulcis*, *Pandorvirus salinus* (Legendre et al., 2018), *Paramecium bursaria* Chlorella Virus 1 (PBCV1) (Dunigan et al., 2012), and Tupanvirus soda lake (Abrahao et al., 2018). Packaged proteins were compared to one another by BLASTp using command line BLAST version 2.8.1+ (Camacho et al., 2009). Reciprocal best BLAST hit (RBH) pairs were determined using bit-score with a maximum e-value cutoff < 1 × 10^–15^. We focused on RBH pairs where proteins were found to be packaged by both viruses. RBH pairs were grouped based on their Nucleocytoplasmic virus orthologous groups (NCVOGs) (Yutin et al., 2009). Clustering of viruses based on the presence/absence of RBH pairs was performed using Bray–Curtis similarity in Primer version 7 (Clarke and Gorley, 2015). To compare clustering by RBH pairs, a maximum likelihood DNA polymerase (PolB) phylogenetic tree was generated using PhyML 3.0 as described previously (Moniruzzaman et al., 2016). RBH pairs were visualized using Circos (Krzywinski et al., 2009).

### Environmental Relevance of Giant Virus Carbohydrate Lyase

We explored data from the TARA Oceans expedition to understand the global diversity of polysaccharide lyases encoded by viruses (Pesant et al., 2015). All the assembled TARA Oceans metagenomic assembled contigs (Supplementary Table 4) were screened for polysaccharide lyases by BLASTx using command line BLAST version 2.8.1+ (Camacho et al., 2009), using an *e*-value cutoff < 1 × 10^–10^. Only biochemically characterized polysaccharide lyases from the Carbohydrate-Active Enzyme (CAZy) Database (Lombard et al., 2014) and those found within the *A. anophagefferens* and AaV genomes were used in our database. To determine if the contigs containing polysaccharide lyases were potentially viral, contigs with polysaccharide lyases were subsequently screened for NCVOGs (Yutin et al., 2009) using BLASTx. A contig was termed viral if it had NCVOG hit with an *e*-value cutoff < 1 × 10^–10^, with the best BLAST hit from the NCVOG BLAST used to assign the contigs putative family. The portion of each of these contigs containing the polysaccharide lyase was excised using a python script (Gann et al., 2019) and then translated into their amino acid sequence. Polysaccharide lyases from viral contigs, which were >80% the length of the smallest biochemically characterized polysaccharide lyase in the database, were placed on a reference maximum likelihood phylogenetic base tree made in PhyML 3.0 using pplacer as described previously (Moniruzzaman et al., 2016). This reference maximum likelihood tree was constructed using the biochemically characterized polysaccharide lyases from the CAZy Database and those found within the *A. anophagefferens* and AaV genomes.

Phylogenetic trees were also created for the NCVOGs present on some of the contigs using PhyML. Contigs from all depths at each station were combined to look at the global distribution of these genes. To determine the relative abundance of contigs containing these polysaccharide lyases and their activity, a single station (Station 122) was used. Reads from the metagenomes (Supplementary Table 4) for which the contigs were assembled were quality filtered and trimmed before being mapped to the entire contig (0.9 identity fraction, 0.9 length fraction) using CLC Workbench version 12.0 (Qiagen, Germany). Reads from metatranscriptomes (Supplementary Table 4) at the same depth of the metagenomes used to assemble each contig were quality filtered and trimmed before being mapped to the polysaccharide lyase of the contig (0.9 identity fraction, 0.9 length fraction) using CLC Workbench version 12.0 (Qiagen, Germany). Reads that mapped to multiple contigs were discarded. One-way ANOVA corrected for multiple comparisons using Tukey’s multiple comparisons was used to assess differences in normalized read abundances using Prism 7.03 (GraphPad, United States).

## Results

### Structural Characterization of AaV Particle by Cryo-EM Reconstruction

**FIGURE 1:**
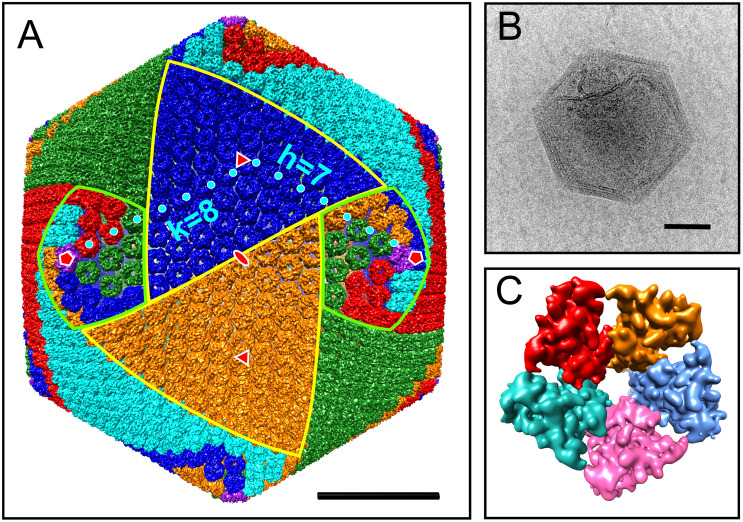
Cryo-EM reconstruction of the AaV capsid. **(A)** Isosurface of AaV capsid. Capsomers are colored based on their orientation in red, blue, green, cyan, and orange. The pentameric capsomers are colored in purple. The borders of two trisymmetrons are highlighted in yellow, whereas the borders of two pentasymmetrons are highlighted in light green. The capsomer centers for calculating *T* number along *h* and *k* axes are labeled by cyan dots. Icosahedral fivefold, threefold, and twofold axes are labeled in red symbols. **(B)** Representative micrograph of an AaV particle. **(C)** Isosurface of the pentameric capsomer. The scale bars in panels **(A,B)** represent 500 Å.

To begin to characterize the particle, the capsid structure was reconstructed. Not only does this provide structural information, but this also provides a means to normalize proteomic data below. In total, 570 selected images of AaV particles were used to produce a 9.5-Å-resolution reconstruction of the 1900-Å viral capsid. At this resolution, individual capsomers were resolved with each composed of three copies of the MCP (Figure 1A and Supplementary Video 1). The reconstructed map of the virus particle does not show any decorations on the outer capsid such as fibers, which is consistent with observation of raw micrographs. Due to a limited number of images, a fivefold-only reconstruction could not be performed to confidently determine whether there is a unique vertex of AaV as seen in PBCV-1 and Mimivirus (Cherrier et al., 2009; Xiao et al., 2009). However, the internal membrane of many particles appeared to be pulling away from the capsid at some vertices (Figure 1B).

All MCPs of NCLDVs have so-called “double jelly-roll” structures with two tandemly linked wedge-shaped β-sandwiches, giving the trimeric capsomer a pseudo-hexameric shape. Besides the trimeric double jelly-roll capsomers, 12 pentameric capsomers located on the vertices of the viral capsid were also clearly resolved (Figure 1C). As seen in other NCLDV members (Klose et al., 2016; Fang et al., 2019; Liu et al., 2019), the pentameric capsomers are made of five copies of an unknown protein (Figure 1C), possibly a single jelly-roll protein. The assembly of icosahedral viruses using a repeating monomer has been described mathematically by the triangulation number, *T* number (Caspar and Klug, 1962). The *T* number can be calculated by counting capsomers along a path following two axes *h* and *k* within the capsid array from one pentamer to the adjacent pentamer (Figure 1A), using the equation *T* = *h*^2^ + *hk* + *k*^2^. With this approach, AaV has a triangulation number of 169, with *h* = 7 and *k* = 8. This is identical to the *T* number of the *Paramecium bursaria* Chlorella Virus, PBCV-1, which has the same diameter of AaV, ∼1900 Å (Fang et al., 2019). The *T* number indicates how many triangle units (e.g., jelly-rolls) are in the asymmetric unit of an icosahedral virus. Therefore, the AaV capsid contains 10,140 (169 × 60) jelly-rolls: 10,080 (168 × 60) are from pseudo-hexameric capsomers (or 5040 double jelly-roll MCP) and 60 are from 12 pentameric capsomers. Interestingly, the *h*-value of 7 is conserved within all icosahedral NCLDVs, with *k* increasing or decreasing based on the *T* number and size of individual particles (Table 1).

**TABLE 1:** Characteristics of structural studies of large icosahedral viruses.

	AaV	PBCV1	PpV-01	Melbournevirus	Pacmanvirus	Faustovirus	CroV
Diameter (nm)	190	190	220	232	250	260	300
*h*	7	7	7	7	7	7	7
*k*	8	8	10	13	13	12	18
*T* number (*T*)	169	169	219	309	309	277	499
Capsomers	1680	1680	2180	3080	3080	2760	4980
MCP copies	5040	5040	6540	9240	9240	8280	14940
Citation	This study	Fang et al., 2019	Yan et al., 2005	Okamoto et al., 2018	Andreani et al., 2017	Klose et al., 2016	Xiao et al., 2017

### Proteomics of Purified AaV Particle

To resolve the tools AaV uses to change the infected cell within 5 min of infection (Moniruzzaman et al., 2018), we determined which proteins are packaged in purified particles. Forty-three virus-encoded proteins were detected in at least one of the three proteomic runs of biological replicates (14 detected in all three runs, 5 detected in only two runs) (Table 2). The most abundant protein detected in all three samples was the MCP (AaV_096) (Supplementary Table 1). With the structural information described above, we estimated the number of proteins per particle. This told us the relative amount of each protein packaged and allowed for the depth of sampling achieved to be determined. Using the mass spectrometry intensities (Supplementary Table 1) and the calculated number of MCP copies per virus particle (5040) from the cryo-EM reconstruction (Table 1), we normalized mass spectrometry intensities to estimate the number of proteins per particle (Supplementary Table 1). According to these predictions, our sampling was representative of proteins packaged within the particle, as we detected proteins that would be present in low abundances (<10 copies per particle) (Supplementary Table 1). For proteins found in multiple samples, the coefficient of variation (CV) was variable between runs (Supplementary Table 1), suggesting that these values can be used only to denote trends. 56 host proteins were also detected in this analysis (Supplementary Table 1), 20 of which were found in multiple samples. Whether some of these proteins are packaged or contamination is unknown, but as none of the host proteins found in all three samples had a CV < 36%, it appears that these may be contamination (Supplementary Table 1), as predicted previously (Legendre et al., 2018).

**TABLE 2:** Characteristics of the 43 proteins packaged within the AaV particle.

Accession number	AaV Gene number	Description	Region of AaV*	MW (kDa)	Transcriptome first detection^†^	Samples found in
**Structural**						
YP_009052173	096	Capsid Protein	Terminal A	52.121	5 min	3
YP_009052321	247	Putative capsid protein 2	Central	64.784	12 h	1
**Transcription**						
YP_009052248	174	DNA directed RNA polymerase K subunit	Central	9.377	6 h	1
YP_009052254	180	Putative VV D6R-type helicase	Central	86.779	12 h	1
YP_009052285	211	Putative mRNA capping enzyme	Central	107.644	5 min	1
YP_009052296	222	Putative DNA directed RNA polymerase II small subunit	Central	126.625	12 h	1
YP_009052316	242	Putative DNA directed RNA polymerase II large subunit	Central	153.787	6 h	1
YP_009052343	269	Putative ATP-dependent RNA helicase	Terminal B	110.004	5 min	1
Polysaccharide degrading/binding						
YP_009052116	038	Pectate lyase	Terminal A	43.978	6 h	3
YP_009052102	024	Concanavalin A-like lectin/glucanase superfamily	Terminal A	231.595	5 min	3
YP_009052156	078	Putative unsaturated glucuronyl hydrolase	Terminal A	55.443	5 min	2
YP_009052348	274	Putative beta-1,4 galactosyltranferase	Terminal B	93.99	5 min	1
YP_009052350	276	Concanavalin A-like lectin/glucanase superfamily	Terminal B	45.07	6 h	3
YP_009052454	386	Concanavalin A-like lectin/glucanase superfamily	Terminal B	60.551	12 h	3
YP_009052388	314	Concanavalin A-like lectin/glucanase superfamily	Terminal B	34.009	6 h	3
**Other**						
YP_009052233	158	Metal-dependent hydrolase	Central	22.941	12 h	2
YP_009052355	281	Putative membrane protein	Terminal B	20.857	12 h	1
YP_009052380	306	Putative ABC transporter family protein	Terminal B	61.006	5 min	1
Hypothetical						
YP_009052130	052	Hypothetical (DUF285 containing)^§^	Terminal A	83.579	5 min	3
YP_009052140	062	Hypothetical	Terminal A	20.844	5 min	3
YP_009052152	074	Hypothetical^§^	Terminal A	11.423	12 h	1
YP_009052158	080	Hypothetical^§^	Terminal A	9.594	5 min	3
YP_009052159	081	Hypothetical^§^	Terminal A	10.239	1 h	1
YP_009052169	091	Hypothetical^§^	Terminal A	42.516	5 min	2
YP_009052180	103	Hypothetical^§^	Terminal A	44.076	1 h	1
YP_009052184	107	Hypothetical^§^	Terminal A	42.877	5 min	1
YP_009052212	136	Hypothetical^§^	Central	45.714	12 h	3
YP_009052220	144	Hypothetical^§^	Central	30.236	30 min	1
YP_009052241	166	Hypothetical	Central	16.066	12 h	1
YP_009052249	175	Hypothetical	Central	34.298	30 min	3
YP_009052256	182	Hypothetical^§^	Central	13.83	12 h	1
YP_009052288	214	Hypothetical (weak BLAST hit to P11, zip protein)^‡^	Central	22.997	30 min	3
YP_009052299	225	Hypothetical^§^	Central	20.592	5 min	2
YP_009052305	231	Hypothetical^§^	Central	15.689	21 h	3
YP_009052306	232	Hypothetical (weak BLAST hit to P2, tape measure)^‡^	Central	57.378	1 h	3
YP_009052324	250	Hypothetical^§^	Central	17.491	12 h	1
YP_009052334	260	Hypothetical^§^	Central	64.878	30 min	1
YP_009052353	279	Hypothetical^§^	Terminal B	28.328	12 h	1
YP_009052360	286	Hypothetical^§^	Terminal B	183.451	5 min	1
YP_009052378	304	Hypothetical^§^	Terminal B	36.271	5 min	1
YP_009052402	328	Hypothetical (BLAST hit to P9, stabilizes pentasymmetrons)^‡^	Terminal B	20.435	12 h	1
YP_009052403	329	Hypothetical^§^	Terminal B	116.31	5 min	1
YP_009052450	382	Hypothetical (DUF285 containing)^§^	Terminal B	35.237	5 h	1

*Region of AaV is based description from the initial characterization of the genome (Moniruzzaman et al., 2014). ^†^Data from the transcriptome of the infection cycle (Moniruzzaman et al., 2018). ^‡^BLAST results against the minor structural proteins determined by the reconstruction of the PBCV-1 particle (Fang et al., 2019). ^§^ Hypothetical proteins that have no BLAST hits in NCBI.

During the genomic analyses of AaV, it was noticed that most of the genes with a predicted NCLDV origin were found in the central part of the linear genome, while many genes predicted to be acquired horizontally were found within the outer thirds and on either side (Moniruzzaman et al., 2014). Genes for packaged proteins are evenly distributed across the genome, with 12, 13, and 18 of the encoded genes found in terminal region A, terminal region B, and the central region, respectively (Supplementary Figure 1A). Eight of the 43 packaged proteins have reciprocal best BLAST hit (RBH) proteins packaged by other NCLDV members (*E*-threshold = 1 × 10^–15^). Interestingly, five of the eight are found within the central region, with two other proteins found just outside this region (Supplementary Figure 1A). The two time points during the infection cycle where the most packaged protein encoding genes were first transcribed were 5 min and 12 h post infection, accounting for 16/43 and 13/43 proteins, respectively, although first detection of transcripts for genes encoding the remaining packaged proteins occurred sporadically over the sampling times (Supplementary Figure 1B) (Moniruzzaman et al., 2018).

### Comparison of Giant Virus Proteomes

**FIGURE 2:**
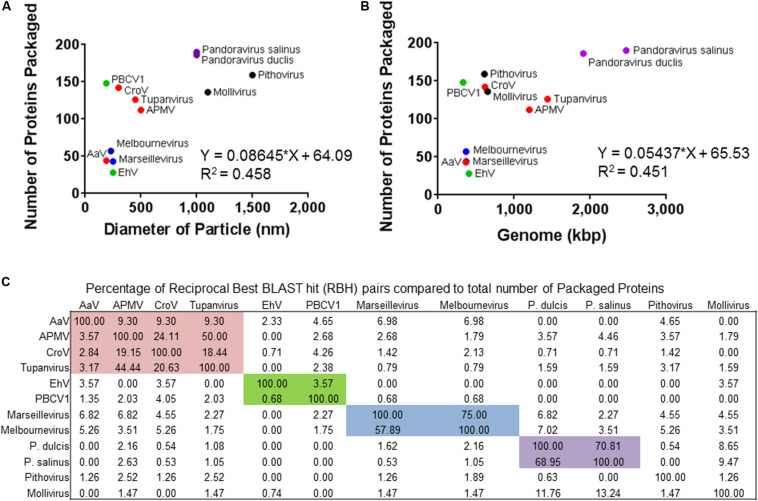
Overview of the other proteomes of packaged proteins used in this study. The number of proteins packaged by each virus was compared to **(A)** diameter of the particle or **(B)** the genome size. **(C)** Percentage of total proteins packaged by the virus (rows) that share a reciprocal best BLAST hit pairs shared between each other (columns). Colored boxes represent the families of viruses. The colors of the icons or boxes indicate viral families with more than one representative: red = *Mimiviridae*, green = *Phycodnaviridae*, blue = *Marseilleviridae*, and purple = *Pandoraviridae*.

**FIGURE 3:**
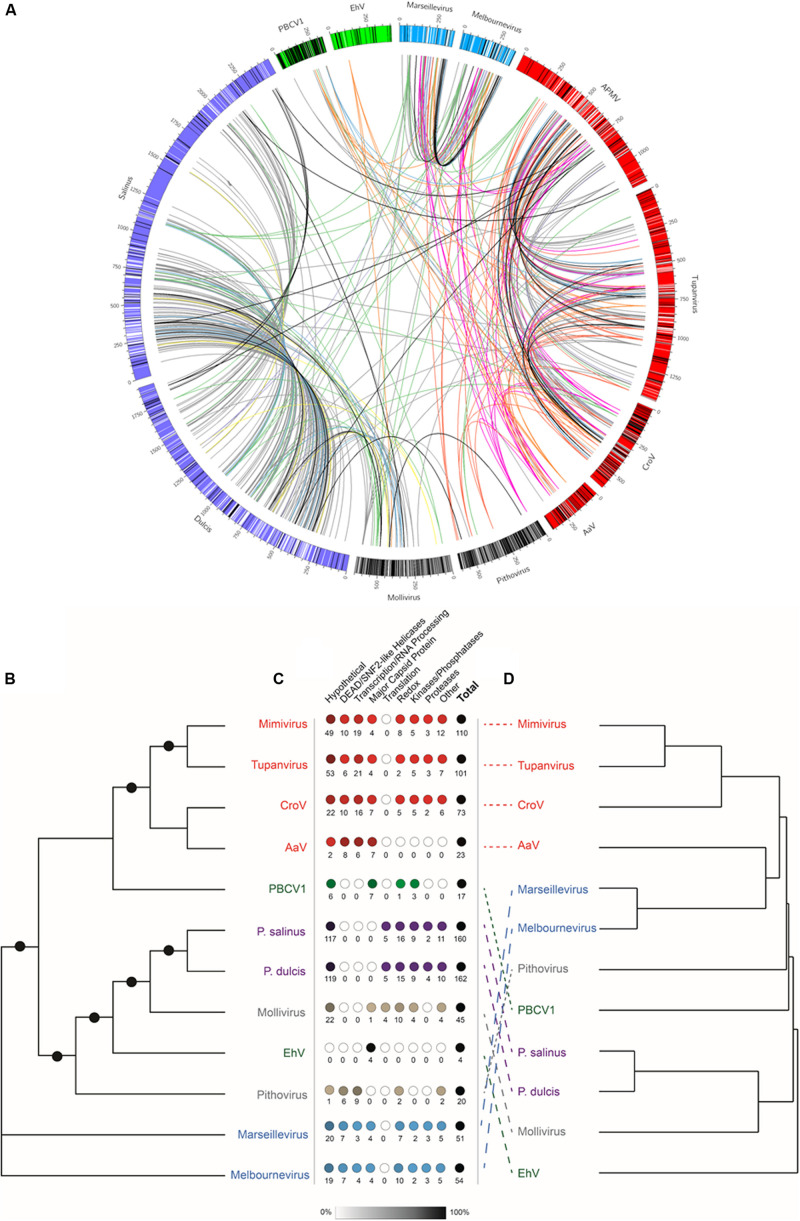
Comparison of packaged proteins between representative viruses. **(A)** The outer bars are the genomes of each of the representative viruses, where there the color is based on the viral family it belongs to: red = *Mimiviridae*, green = *Phycodnaviridae*, blue = *Marseilleviridae*, and purple = *Pandoraviridae*. The black lines represent the location of the genes encoding proteins packaged in the particle that do not have reciprocal best BLAST hits to any of the other representatives, while white bare lines represent the location of the genes encoding proteins packaged in the particle that do have reciprocal best BLAST hits to any of the other representatives. The lines between viruses are connecting reciprocal best BLAST hit pairs with their color being based on the classification of the NCVOG category (Supplementary Table 3). The colors are as follows: uncharacterized pairs = gray, DEAD/SNF2-like helicases = pink, Redox = green, transcription/RNA processing = red, translation = yellow, capsid proteins = orange, proteases = purple, kinases/phosphatases = blue, other = black. **(B)** Unrooted maximum likelihood DNA Polymerase B (PolB) phylogenetic tree. Node support (aLRT-SH statistic) > 50% are shown as dark circles. **(C)** Number of reciprocal best BLAST hit pairs found within each virus proteome by type of NCVOG category (Supplementary Table 3). Numbers below the circles indicate the number of reciprocal best BLAST hit pairs. Shading of the circles represent the percentage of the total number of reciprocal best BLAST hit pairs. **(D)** Hierarchical clustering of resemblance based on Bray–Curtis similarity of presence or absence of reciprocal best BLAST hit pairs for all proteins found within the representative particle proteomes. The colors of the virus names indicate viral families with more than one representative: red = *Mimiviridae*, green = *Phycodnaviridae*, blue = *Marseilleviridae*, and purple = *Pandoraviridae*.

Given the large number of NCLDV particles with characterized proteomes (Figure 2), we examined similarities and differences between virus families regarding proteins packaged. Of the proteomes considered, there was a correlation between number of proteins packaged and both particle diameter [Figure 2A, Packaged Proteins = 0.086^∗^(particle diameter) + 64.09, *R*^2^ = 0.458] and genome size [Figure 2B, Packaged Proteins = 0.054^∗^(genome size) + 65.53, *R*^2^ = 0.451], although the number of packaged proteins across these viruses varied from 28 (Allen et al., 2008) to ∼200 (Legendre et al., 2018). RBH pairs of all proteins between representative genomes were determined, and RBH pairs with both proteins in the pair packaged by each virus were examined (*e*-value cutoff < 1 × 10^–15^). Of the 1,367 proteins packaged, there were a total of 410 RBH pairs (Figure 3A and Supplementary Data 1.1), which were classified based on their NCVOG classifications (Supplementary Table 2 and Supplementary Data 1.1) (Yutin et al., 2009). Viruses in some families (i.e., *Pandoraviridae* and *Marseilleviridae*) appear to package many similar proteins, while other families do not (i.e., *Phycodnaviridae*) (Figures 2C, 3C). Of the 410 RBH pairs, only 119 were shared between families (Supplementary Table 2). Hierarchical clustering based on similar proteins revealed similar groupings to those determined phylogenetically (Figures 3B,D).

### Virus-Encoded Polysaccharide-Degrading Enzymes in the TARA Oceans Dataset

The particle packages multiple proteins involved in sugar metabolism, including multiple with sugar binding motifs, and two predicted to degrade polysaccharides (AaV_038 and AaV_078) (Table 2). We were interested in the pectate lyase that is packaged (AaV_038), as there are three encoded in the genome of AaV, all believed to be acquired horizontally (Moniruzzaman et al., 2014). Re-examining host and viral polysaccharide lyases over the infection cycle compared to control in the transcriptomics data, we determined that host lyase transcript abundance remains constant, but the viral lyase abundance greatly increases (Supplementary Figure 1C), suggesting their importance in the infection cycle. As genes encoding sugar degradation proteins have been found in other algal viruses (Van Etten et al., 2017), but have not yet been found to be packaged, we wanted to further look at the distribution of these virally encoded polysaccharide lyases, using the TARA Oceans dataset.

The biochemically characterized polysaccharide lyases in the CAZy Database (Lombard et al., 2014) degrade a wide variety of polysaccharide polymers (Supplementary Figure 2). We searched all assembled contigs from all metagenomes from the TARA Oceans dataset using these biochemically characterized proteins and found 11,055 contigs with a near full-length polysaccharide lyase present (Supplementary Data 1.2–1.4). Searches for NCVOGs on these contigs revealed that 499 could be classified as potentially of viral origin. Using the best BLAST hit on the NCVOGs marker, these contigs were assigned to eight viral families, with the majority belonging to either *Phycodnaviridae* (183/499) or *Mimiviridae* (142/499) (Supplementary Table 3). As NCLDVs range in size from <0.22 to >1.5 μm in diameter, using all the metagenomes was warranted instead of focusing on a single fraction size. Increased confidence that these contigs are viral in origin stems from there being no difference in the percentage of the total polysaccharide lyases that come from putative viral contigs in metagenomes by sampling depth (Supplementary Figure 3A and Supplementary Data 1.2, 1.5). There is, however, a decrease in this percentage based on the size fraction of the water used in sample collection (Supplementary Figure 3B and Supplementary Data 1.3, 1.6).

**FIGURE 4:**
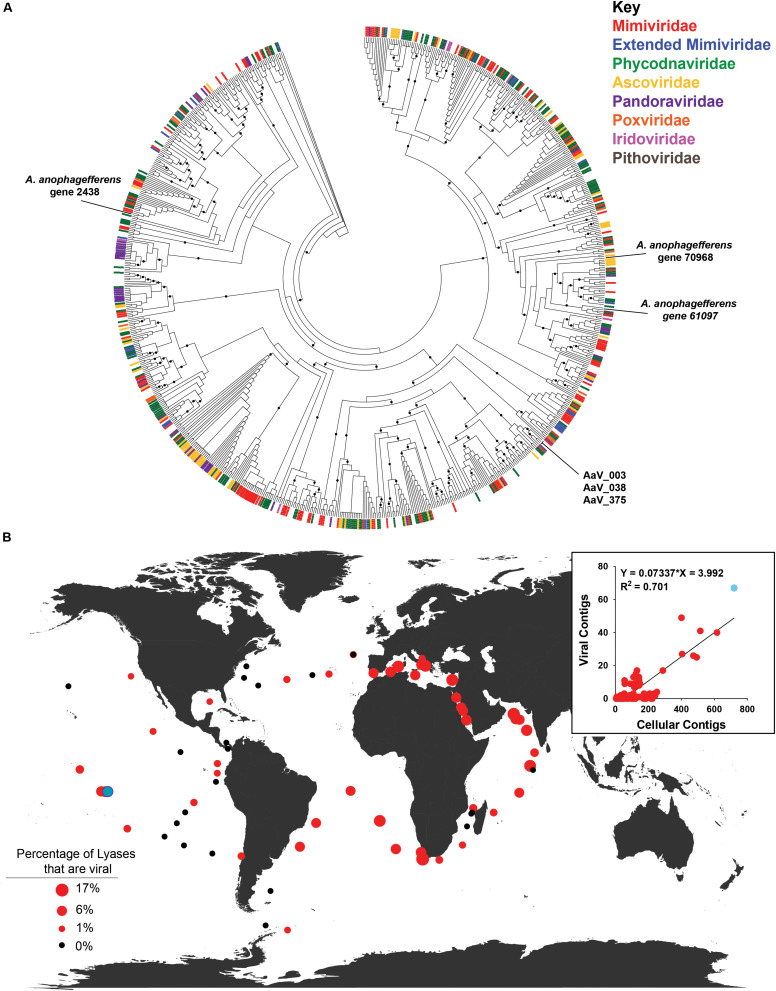
Distribution of viral contigs containing polysaccharide lyases. **(A)** Maximum-likelihood phylogenetic placement of polysaccharide lyases from putative viral TARA Oceans metagenomic contigs. The classification of the contig by family based on the NCVOG on the contig are denoted by color bar. Reference-characterized polysaccharide lyases from the Carbohydrate-Active Enzyme (CAZy) Database do not have a bar at the end of their branch. Node support (aLRT-SH statistic) > 50% are shown as dark circles. **(B)** Contribution of polysaccharide lyases found on viral contigs to total contigs containing polysaccharide lyases for all depths at each TARA Oceans station. Black dots represent stations where no viral contigs containing polysaccharide lyases were found. Inset: TARA Oceans metagenomic contigs containing polysaccharide lyases by station. Station 122 is colored in light blue.

Placing the 499 polysaccharide lyases from contigs of viral origin onto the base tree of characterized pectate lyases (Supplementary Figure 2) using pplacer revealed no phylogenetic grouping of these contigs by assigned viral family (Figure 4A). Five polysaccharide lyases from viral contigs from three stations clustered with the three AaV pectate lyases (Supplementary Figure 4A). As three of the NCVOGs used to call those viral contigs were present in the AaV genome, phylogenetic trees were constructed to determine if closely related polysaccharide lyases would be from phylogenetically related viruses (Supplementary Figures 4B–D). Two of the three contigs cluster with AaV (Supplementary Figures 4B,C), while the other clusters between the *Mimiviridae* and Extended *Mimiviridae* (Supplementary Figure 4D). The distribution of viral contigs containing polysaccharide lyases was correlated [viral lyase contigs = 0.07337 (non-viral lyase contigs) − 3.992, *R*^2^ = 0.701] with non-viral contigs containing polysaccharide lyases (Figure 4B, inset). At some stations, up to 17% of polysaccharide lyases detected were putative contigs of viral origin (Figure 4B). Using a single station as an example (Station 122, Figure 4B), read mappings of the metatranscriptomes and metagenomes to contigs were as proxies for activity of the polysaccharide lyases and the abundance of the contigs. This station was chosen as it had the highest abundance of polysaccharide lyases containing contigs and polysaccharide lyases on viral contigs (Supplementary Data 1.4 and Figure 4B, inset). In two of the three water depths, there was no significant difference between the metagenomic reads mapped back to contigs between viral and non-viral contigs containing polysaccharide lyases, while in surface waters, there were slightly but significantly more reads mapped to viral contigs containing polysaccharide lyases (Supplementary Figure 3C and Supplementary Data 1.7). There were no significant differences in the expression between the viral and non-viral polysaccharide lyases at any water depth (Supplementary Figure 3D and Supplementary Data 1.8).

## Discussion

The goal of this study was to quantitatively determine the protein ensemble within an average AaV particle using proteomics. To normalize the data to individual particle complements, we coupled this work with a cryo-electron microscopy reconstruction of the virus particle to determine the protein distribution per particle of a significant marker protein (the MCP). We determined that the structure, assembly, and some proteins packaged within the AaV particle are conserved with other giant virus particles, yet some are unique to cultured representatives. We then looked for signatures of polysaccharide lyases encoded by viruses in the oceans as at least one of three putative polysaccharide lyases are packaged in the AaV particle. We found many of these genes virally encoded in many distinct locations throughout the world. Collectively our observations demonstrate that giant virus particles have the potential to carry proteins that may be functional metabolically and that these proteins are broadly distributed in the world’s oceans.

### Structural Characteristics of the AaV Particle

The structure of the external surface of large icosahedral viruses is variable (Xiao et al., 2017). Many viruses possess fibrils, including Mimivirus with 1250-Å-long fibers, or *Megavirus chilensis* with smaller 750-Å-long fibers (Claverie et al., 2009; Arslan et al., 2011), and multiple of these can bind to one subunit of the capsid forming a densely packed network. External fibrils have been hypothesized to have different roles, including stabilizing the capsid after adsorption (Zhang et al., 2011), and helping viral attachment to their host and other organisms to aid in dissemination (Rodrigues et al., 2015). Although fibrils are found on the surface of viruses from diverse families within the NCLDVs, not possessing fibrils is also not an unique phenotype (Xiao et al., 2017; Okamoto et al., 2018). As shown in the micrograph in Figure 1B, the AaV particle does not appear to have fibrils on the outside of its capsid.

Like other icosahedral viruses, with the exception of the Faustovirus (Klose et al., 2016), AaV possesses an electron-dense core surrounded by a putative membrane. While the mechanism of entry of AaV into *A. anophagefferens* is unknown, the putative membrane surrounding the core allows for the fusion of the host and virus membrane as seen in other systems (Zauberman et al., 2008; Milrot et al., 2017). Interestingly, this internal membrane does not appear to be maintained at a uniform distance from the capsid, as pockets were observed beneath some vertices. While no unique physical structures were detected with the current reconstruction due to the limitation of image numbers, these larger pockets could serve to store proteins essential to the initial stages of the infection. A similar space was previously observed in Mimivirus particle beneath its star-gate unique portal (Xiao et al., 2009). In Mimivirus, the opening of this unique portal allows for fusion of the viral and phagosome membranes (Zauberman et al., 2008). This has also been observed in the PBCV-1 particle, where an unique spike vertex contains a pocket below that is hypothesized to have the enzymes required for the degradation of the cell wall of *Chlorella* NC64A (Cherrier et al., 2009). To this end, it may be that an internal membrane structure is as important as surface decorations with respect to the process of infection.

### Virus Particle Reconstruction

The 9.5-Å-resolution reconstruction of the 1900-Å viral capsid allowed for the determination of individual capsomers and their orientation. The arrangement of capsomers into distinct sub-capsid structures, i.e., symmetrons, were first described in the late 1960s in a structural study of *Sericesthis* iridescent virus (SIV) (Wrigley, 1969) when pentagonal and triangular patches of capsomer arrays were observed in SIV degradation samples. These sub-capsid structures are denoted trisymmetrons and pentasymmetrons. Geometrically, they cover the 20 threefold faces and 12 fivefold vertices of icosahedral viruses. The trisymmetrons of AaV consist of 66 trimeric capsomers that all face the same orientation, 60° rotated to the adjacent trisymmetron and pentasymmetrons. The pentasymmetron of AaV consists of 30 hexameric capsomers and 1 pentameric capsomer, made from an unknown protein. The size of AaV pentasymmetron is the same as all icosahedral NCLDVs. The orientation of these capsomers within a pentasymmetron has been discussed previously (Xiao et al., 2017; Okamoto et al., 2018). A spiraling mechanism for assembly was first proposed for CroV, which states that the trisymmetron is built continuously from the pentasymmetron from a uniquely oriented capsomer, which seeds the position of all the capsomers within the trisymmetron (Xiao et al., 2017). Yet, due to the similarities between the sub-capsid structure of various NCLDV members, it is believed that they all are assembled in a similar manner, including AaV.

### Structural Proteins Detected in the AaV Particle

Proteomics performed on purified AaV particles detected at least 43 proteins, with the most abundant protein detected being the MCP. This is less than other members of the Mimiviridae with CroV, Tupanvirus, and Mimivirus packaging 141, 127, and 114 (reanalyzed to be 137) viral encoded genes, respectively (Renesto et al., 2006; Claverie et al., 2009; Fischer et al., 2014; Abrahao et al., 2018). Although any heterogeneity of proteins packaged may be lost by bulk analysis, proteomics provides information on the average AaV particle. Using the structural information determined by cryo-EM, we were able to assess the number of proteins per particle. In addition to the MCP, CroV possesses three other capsid proteins that have a similar molecular weight to the CroV MCP. Among these capsid proteins is capsid protein 2 that is predicted to be in 60 copies per particle, and capsid proteins 3 and 4 that are in only 1 or 2 copies per particle, respectively. It is hypothesized that, together with two MCPs, the capsid protein 2 form a heterotrimeric capsomer, which is located in the pentasymmetron with a unique orientation to the other capsomers in the same pentasymmetron asymmetric unit (Xiao et al., 2017). Therefore, the capsid protein 2 could be involved in the assembly of the viral particle. Due to the low abundance per particle, the other two proteins are hypothesized to be involved in forming a putative unique vertex structure of CroV (Xiao et al., 2017). Besides the MCP, only one other putative capsid protein (AaV_247) is encoded by the genome of AaV (Moniruzzaman et al., 2014), and that was detected in only one sample. The AaV_247 protein appears to play a very minor role in the structure, as there are only seven copies per particle when normalized by the MCP in that single run. It is possible that this capsid protein may be involved in the assembly of a unique vertex, although the presence of a unique vertex of AaV cannot be confirmed in this study.

PBCV-1 also encodes multiple minor structural proteins (mCPs) that form an intensive network underneath the MCP and may play a role in stabilizing capsid structure. A network similar to this was also seen in ASFV (Liu et al., 2019). While 13 different types of mCPs were found in PBCV-1, only three types were identified in ASFV. Through BLASTp searches against mCPs within the PBCV-1 particle (Fang et al., 2019), three putative mCPs were found within the AaV particle. AaV_328 has a BLASTp hit (Query Coverage 88%, *e*-value 6 × 10^–19^) to the PBCV-1 P9 protein (A407L). P9 is located beneath the MCPs within the pentasymmetrons and predicted to stabilize the pentasymmetrons and its association with the inner viral membrane (Fang et al., 2019). AaV_214 (detected in all three runs) also had a weak BLASTp hit (Query coverage: 26%, *e*-value: 1 × 10^–9^) to the mCP P11, which is named the zip protein and is hypothesized to help join neighboring trisymmetrons (Fang et al., 2019). Finally, AaV_232 had a very weak hit to the PBCV-1 tape measure protein P2 (Query Coverage: 21%, *e*-value: 0.006). When BLASTp searched against the ASFV tape measure protein (M1249L), and the predicted tape measure proteins in Mimivirus (*L454*), and CroV (*crov185*), AaV_232 only weakly hit to L454 (Query Coverage: 13%, *e*-value: 5 × 10^–10^) and did not hit the CroV or ASFV proteins with significant coverage. That said, the length of the tape measure protein (TmP) is hypothesized to be correlated with the size of the virus. AaV and PBCV-1 have the capsids with the same diameter (1900 Å): AaV’s putative TmP consists of 505 amino acids, similar to the PBCV-1 TmP (576 amino acids). The Mimivirus putative TmP has 1257 amino acids, which is consistent with the length predicted by PBCV-1 TmP (Fang et al., 2019). The presence of TmP among all four giant viruses mentioned above emphasizes its important role in determining the size as well as the assembly of the viral capsid. Although not all the mCPs found in PBCV-1 were detected in the AaV particle, these findings suggest that these mCPs play roles in viral capsid assembly and stability. The high-resolution structure of the AaV virion will confirm these observations.

### Transcriptional Machinery Detected in the AaV Particle

The remaining six proteins found in other NCLDV particles are predicted to be involved in transcription. Transcription-related proteins make up 14% of the proteins packaged in AaV, similar to other *Mimiviridae* members where 10.5% (12/114), 11.1% (16/141), and 10.2% (13/127) packaged are transcription related in Mimivirus, CroV, and Tupanvirus soda lake, respectively (Renesto et al., 2006; Fischer et al., 2014; Abrahao et al., 2018). Unlike Mimivirus, which packages all the necessary subunits of RNA polymerase for transcription (Renesto et al., 2006), AaV appears to be more reliant on the host, as we detected only three subunits of RNA polymerase within the AaV particle (AaV_174, AaV_222, and AaV_242). AaV_242 and AaV_222 are similar to the two largest and active subunits (J6R and A24R) of the Vaccinia virus RNA polymerase capable of transcription of the early genes *in vivo* and *in vitro* (Broyles, 2003). Other transcriptional machinery within the AaV particle included two putative RNA helicases (AaV_180, AaV_269) and a putative mRNA capping enzyme (AaV_211), which are found in the other *Mimiviridae* proteomes (Renesto et al., 2006; Fischer et al., 2014; Abrahao et al., 2018). While the mechanism of how AaV infects its host remains unknown, it is likely different from the phagocytosis seen with the other larger members of the *Mimiviridae* (Mutsafi et al., 2010; Fischer et al., 2014; Abrahao et al., 2018), which may be why AaV does not need to package all of the components to begin transcription. This observation may highlight an important difference between the algae-infecting Extended *Mimiviridae* and phagocytic-eukaryote infecting *Mimiviridae* members. Yet even without packaged transcriptional machinery (Dunigan et al., 2012), virus transcripts are detected within 7 min post infection for a PBCV-1 infection (Blanc et al., 2014), relying on condensed injected viral DNA being translocated into the nucleus (Milrot et al., 2017). More work is needed to understand the beginning of the AaV infection cycle, as viral transcripts were detected 5 min post infection (Moniruzzaman et al., 2018), suggesting that the genome can also translocate to the nucleus quickly. Alternatively, some mRNA transcripts may be packaged, as this has been shown in other large dsDNA viruses (Boyer et al., 2009).

### Proteins Unique to the AaV Particle

Thirty-two proteins in AaV are not detected in other NCLDV particles. These include a putative metal dependent hydrolase (AaV_158) and an MdlB domain-containing putative ABC transporter (AaV_306), both of which have not been detected in other virus particles yet have BLAST hits to various families within the NCLDVs. Also present are 23 proteins with no putative function, including 19 that do not have any BLAST hits to NCLDV members. AaV_052 and AaV_382 both contain a domain of unknown function (DUF285). These regions are noteworthy as the DUF285 region is contained in 13.25% of the AaV coding regions and found in 90 different locations within the *A. anophagefferens* genome (Moniruzzaman et al., 2014). AaV_175 and AaV_281 have BLAST hits to other NCLDV members, but no putative function.

Of the remaining packaged proteins unique to the AaV particle, all are hypothesized to be involved in sugar metabolism or sugar binding. Four of these proteins (AaV_024, AaV_276, AaV_314, and AaV_386) are Laminin G domain-containing proteins. In total, AaV has five of these paralogs within its genome. This group of genes is unique to AaV in the NCLDV group and are believed to have been acquired horizontally from bacteria (Moniruzzaman et al., 2014). Laminin G domains have been shown to interact with various polysaccharides including heparin (Aumailley, 2013). There are no other domains of known function in any of these proteins so their roles in the infection cycle are still unknown. AaV also packages a putative beta-1,4 galactosyltransferase (AaV_274). Similar proteins in chloroviruses are predicted to synthesize the unique glycans decorating the MCP (Van Etten et al., 2017). It is unknown whether the MCP of AaV is decorated with sugars; however, AaV_274 has BLAST hits to many members of *Iridoviridae* and *Ascoviridae*, suggesting that the role of this protein may not be unique. Packaged within the particle are also two proteins with putative functions involved in degrading polysaccharides not found in other NCLDV members. AaV_078 is a putative unsaturated glucuronyl hydrolase that was likely acquired from *A. anophagefferens*, which possesses a similar protein (AURANDRAFT_70832). Also packaged is one of three putative pectate lyases found within the genome, AaV_038 (Moniruzzaman et al., 2014). These enzymes may play a role in viral DNA entering *A. anophagefferens*, as has been proposed for PBCV-1 (Van Etten et al., 2017), though it is unknown whether AaV injects its DNA into *A. anophagefferens* in a similar manner to PBCV1 (Milrot et al., 2017). However, *A. anophagefferens* cells do lose their outer polysaccharide glycocalyx during the infection cycle. This has been observed in AaV infection in the laboratory (Rowe et al., 2008) as well as in nature when host populations are infected with large number of viruses during blooms (Gastrich et al., 2004). This suggests that sugar metabolism is important during infection. More work is needed to determine the composition of the glycocalyx and the mechanism by which it is degraded.

### Comparison of NCLDV Proteomes

Although the genetic repertoire of these NCLDVs is different within and between viral families, this study supports the contention that the structure and assembly of large icosahedral viruses are evolutionarily conserved. Using the MCP, we were able to normalize and accurately determine protein copies packed in the 1,900-Å particle. We found that AaV packages a large number of proteins that might be important in the degradation of the host polysaccharides and in modulating the host’s capacity to transcribe viral genes. Several of these transcription-related packaged proteins appear to be conserved in the *Mimiviridae* (see section below). Looking at protein packaged across all the NCLDV families, it appears that ubiquitous stresses on viral reproduction, such as oxidative stress and transcription, constrain viruses to package similarly, even if they infect very different hosts or belong to different families.

As there is a growing amount of information about proteins packaged in NCLDVs, we compared the proteins packaged using RBH pairs. *Pandoraviridae* members have previously been shown to package a large number of similar proteins (Legendre et al., 2018). Approximately, 70% of the packaged proteins detected in this analysis are similar between *Pandoravirus salinus* and *Pandoravirus dulcis*. This trend is seen in *Marseilleviridae* members as well (Fabre et al., 2017), but not the two *Phycodnaviridae* members with their packaged proteins determined, with the only RBH pair being the MCP. The more similar the viral host in *Mimiviridae* members, the higher the number of shared packaged proteins between those viruses. *Acanthamoeba* infecting Mimivirus and Tupanvirus have RBH pairs for ∼50% of their packaged proteins. Although CroV infects a different phagocytic host, *Cafeteria roenbergensis* (Fischer et al., 2014), it shares ∼20% of its packaged proteins with Mimivirus and Tupanvirus. This number decreases dramatically to <10% of shared proteins between AaV and the other three members of the *Mimiviridae* family. The autotrophic *A. anophagefferens* has the ability to take up organic nutrients in the environment (Gobler and Sunda, 2012), but it has never been described to have a phagocytic life cycle. This continuum of percentage of similar proteins may suggest that viruses infecting similar hosts must overcome similar challenges within the virocell, while those that infect drastically different hosts do not.

Although the majority of RBH pairs were shared within viral families (291/410), there were many shared between families. However, those shared between families only made up a small portion of the packaged proteins. Approximately 10% of proteins packaged within the Mollivirus particle have a RBH pair with both pandoraviruses, which mirrors the genomic analysis of Mollivirus having the most best-matching homologs (15.9%) to *Pandoravirus salinus* (Legendre et al., 2015). These two families are the only two to package proteins involved in modulating translation of their hosts. Less than 8% of proteins packaged within any of the other representatives have RBH pairs in other families. Besides proteins of unknown function, the 26 RBH pairs involved in redox reactions had the most of any category of RBH pair and are found in all of the representative viruses except for AaV and EhV. Surviving oxidative stress within the host cell and still being able to produce progeny is a universal challenge that needs to be overcome by viruses. This can be seen as increasing oxidative stress has been shown to increase the amount of time for viral factory formation in Mimivirus (Yaakov et al., 2019) and in transcriptomes of infection cycles (i.e., Moniruzzaman et al., 2018). Packaging these proteins can provide an initial counter to the ROS induction that occurs during initial stages of infection. Besides the MCP being shared between many viruses, the only other group of proteins with more than 10 RBH pairs between families are involved in transcription and RNA processing, including DEAD/SNF2-like helicases. These are shared only between members of the *Marseilleviridae*, *Mimiviridae*, and *Pithoviridae*. Even if the viruses are not complete independent of the host’s nucleus (Fabre et al., 2017), there still needs to be a rapid reprogramming of the cell for the infection to proceed, as seen in other systems (Blanc et al., 2014; Moniruzzaman et al., 2018). The similarities and differences of proteins that are packaged between families may provide a glimpse of viruses evolving to survive a range of environments within their host.

### Polysaccharide-Degrading Enzymes in Marine Virus Communities

Given that chloroviruses (Van Etten et al., 2017) encode genes that degrade host polysaccharide polymers and AaV packages a putative pectate lyase, we wanted to see how prevalent these genes were in aquatic environments using the TARA Oceans metagenomic assembled contigs (Pesant et al., 2015). Previously, phylogenetic predictions suggested that AaV acquired the three pectate lyases in its genome from either bacteria or *A. anophagefferens* (Moniruzzaman et al., 2014), based on a subset of similar proteins detected by BLAST. Phylogenetic examination of the polysaccharide lyases found in the CAZy database suggests that AaV did not acquire these proteins from its host. The three proteins within the AaV genome all cluster within a group of pectate lyases, although these polysaccharide lyases do not cluster based on source (i.e., eukaryotes or prokaryotes), or enzyme substrate. Based on the co-occurring NCVOG genes on contigs, most virus-encoded polysaccharide lyases appear to be members of either the *Mimiviridae* or *Phycodnaviridae* families. The ability to break down large polysaccharide polymers is found in phylogenetically distinct groups of bacteria in aquatic communities (Alderkamp et al., 2007), supporting the potential acquisition of these genes by NCLDV from distinct groups. The reason why certain TARA stations were hotspots for polysaccharide lyase presence (viral or non-viral) is unknown, but it is possible that blooms of algae could have occurred. During a *Phaeocystis* spp. bloom, high proportions of the community (up to ∼33%) have been shown to contain enzymes to degrade its polysaccharide storage molecule, laminarin (Alderkamp et al., 2007).

It is becoming apparent that enzymes that degrade large polysaccharides in the environment are not just a product of cells. Virus-encoded or virocell-produced enzymes provide a mechanism to degrade polysaccharides that can persist in the microbial pool of the oceans (i.e., released upon lysis or packaged within virus particles). As not all members of any given microbial community can degrade high-molecular-weight polysaccharides (Muhlenbruch et al., 2018), and members differ in their ability to assimilate dissolved organic matter (Elifantz et al., 2007), enzymes that provide easier-to-consume intermediates may benefit or even stimulate osmotrophs. As it is hypothesized, these intermediates would only be found when there are large amounts of the substrate (Elifantz et al., 2007), and virocell-produced enzymes may especially be important in bloom conditions. Coupling hypothesized virus-mediated bloom collapse (Gastrich et al., 2004; Vardi et al., 2012), with an ability of infection or free-virus particles to modulate extracellular polysaccharide production (Vardi et al., 2012), would provide a large amount of substrate for enzymes to aid in the uptake of carbon for the heterotrophic community during bloom collapse. The observation made here suggests that surveys of metabolic capabilities of microbial community members need to look beyond cellular organisms: viruses not only alter host cell metabolism but may themselves be repositories of active metabolic capabilities that need consideration.

## Data Availability Statement

All accession numbers used in this study are found in Supplementary Table 4, and all raw mass spectrometry intensities are found in Supplementary Table 1.

## Author Contributions

EG and SW designed the experiments. EG generated all samples for cryo-EM and proteomics. Cryo-EM sampling and analysis were performed by YX and CX. Proteomic measurements were made by PA and RH. Bioinformatic analyses were performed by EG. Manuscript was drafted by EG and SW, with all authors contributing to revisions.

## Conflict of Interest

The authors declare that the research was conducted in the absence of any commercial or financial relationships that could be construed as a potential conflict of interest.
