# Supplementary material for: Structural and Proteomic Studies of the Aureococcus anophagefferens Virus Demonstrate a Global Distribution of Virus-Encoded Carbohydrate Processing
Source: Front Microbiol. 2020 Sep 8;11:2047. doi: 10.3389/fmicb.2020.02047 (PMC7507832; doi:10.3389/fmicb.2020.02047)
Supplement: Supplementary file 1 [file Image_1.pdf]

Supplemental information to be published online in conjunction with the following:

Structural and proteomic studies of the Aureococcus anophagefferens Virus demonstrate a global distribution of virus-encoded carbohydrate processing

Eric R. Gann[1†], Yuejiao Xian[2†], Paul E. Abraham[3], Robert L. Hettich[3], Todd B. Reynolds[1], Chuan Xiao[2], and Steven W. Wilhelm[1*]

[1]Microbiology Department, University of Tennessee, Knoxville, TN 37996-1937 USA
[2]Department of Chemistry and Biochemistry, University of Texas at El Paso, El Paso, TX 79968 USA
[3]Chemical Sciences Division, Oak Ridge National Laboratory, Oak Ridge, TN 37831 USA

[†]These authors contributed equally to this work.

*** Correspondence:**
Steven W. Wilhelm, wilhelm@utk.edu, Telephone: 1-865-974-0665 Fax: 1-865-974-4007

Chuan Xiao, cxiao@utep.edu, Telephone: 1-915-747-8657 Fax: 1-915-747-5996

Keywords: Giant Viruses, Viro-cell Metabolism, Proteomics, Cryo-EM, Carbohydrate lyases

Supplemental Movie 1. Movie of 9.5Å resolution reconstruction of the AaV particle.

Supplemental Data 1. Other data from this study. Supplemental Data includes reciprocal best BLAST hit pairs, description of contigs used in this analysis, and p-value tables generated in this study. Data is separated into tabs denoted as the following:

Supplemental Data 1.1: List of reciprocal best BLAST hit pairs with their NCLDV COG category.

Supplemental Data 1.2. Metagenomic assembled contigs from TARA Oceans Dataset separated by water depth.

Supplemental Data 1.3. Metagenomic assembled contigs from TARA Oceans Dataset separated by size fraction.

Supplemental Data 1.4. Metagenomic assembled contigs from TARA Oceans Dataset separated by Station.

Supplemental Data 1.5. One-way ANOVA corrected for multiple comparisons using Tukey's multiple comparisons p-values for percentage of contigs that are viral by water depth.

Supplemental Data 1.6. One-way ANOVA corrected for multiple comparisons using Tukey's multiple comparisons p-values for percentage of contigs that are viral by water fraction.

Supplemental Data 1.7. One-way ANOVA corrected for multiple comparisons using Tukey's multiple comparisons p-values for normalized metagenomics reads.

Supplemental Data 1.8. One-way ANOVA corrected for multiple comparisons using Tukey's multiple comparisons p-values for normalized metatranscriptomics reads.

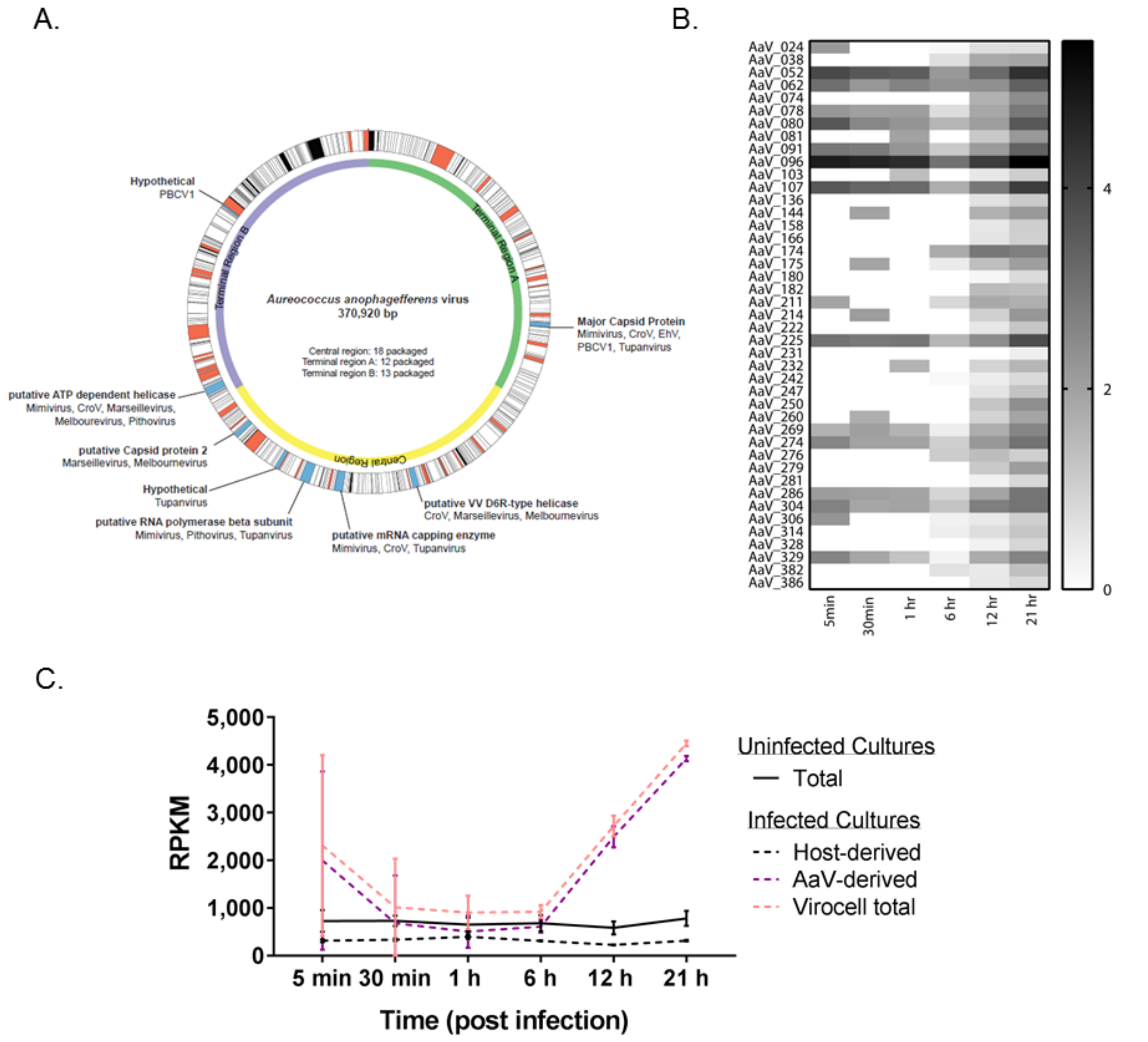

Supplemental Figure 1. Insight into this study from previous genomic and transcriptomic analyses from Moniruzzaman *et al.* 2014 and Moniruzzaman *et al.* 2018. A) Circos plot of the entire AaV genome. The outer ring is decorated with coding sequences within the genome, where white bars are gene products not packaged, red bars are those packaged but that do not have reciprocal best BLAST hits (e value cutoff < 1 x 10^{-15}), and blue bars are gene products packaged and have reciprocal best BLAST hits in representative proteomes used in this study. Those proteins are labelled and have the viruses for which has an RBH hit. The inner ring shows the three segments of the genome described previously (Moniruzzaman et al., 2014). B) Heatmap of the log-transformed RPKM values + 1 of the genes in which the gene products are found within the particle over the course of the infection cycle (Moniruzzaman et al., 2018). C) Combined RPKM values of polysaccharide lyases over the 21-hour infection cycle. The *A. anophagefferens* proteins used were AURANDRAFT_70968, AURANDRAFT_61097, and AURANDRAFT_2438, and the AaV proteins used were AaV_003, AaV_038, AaV_375.

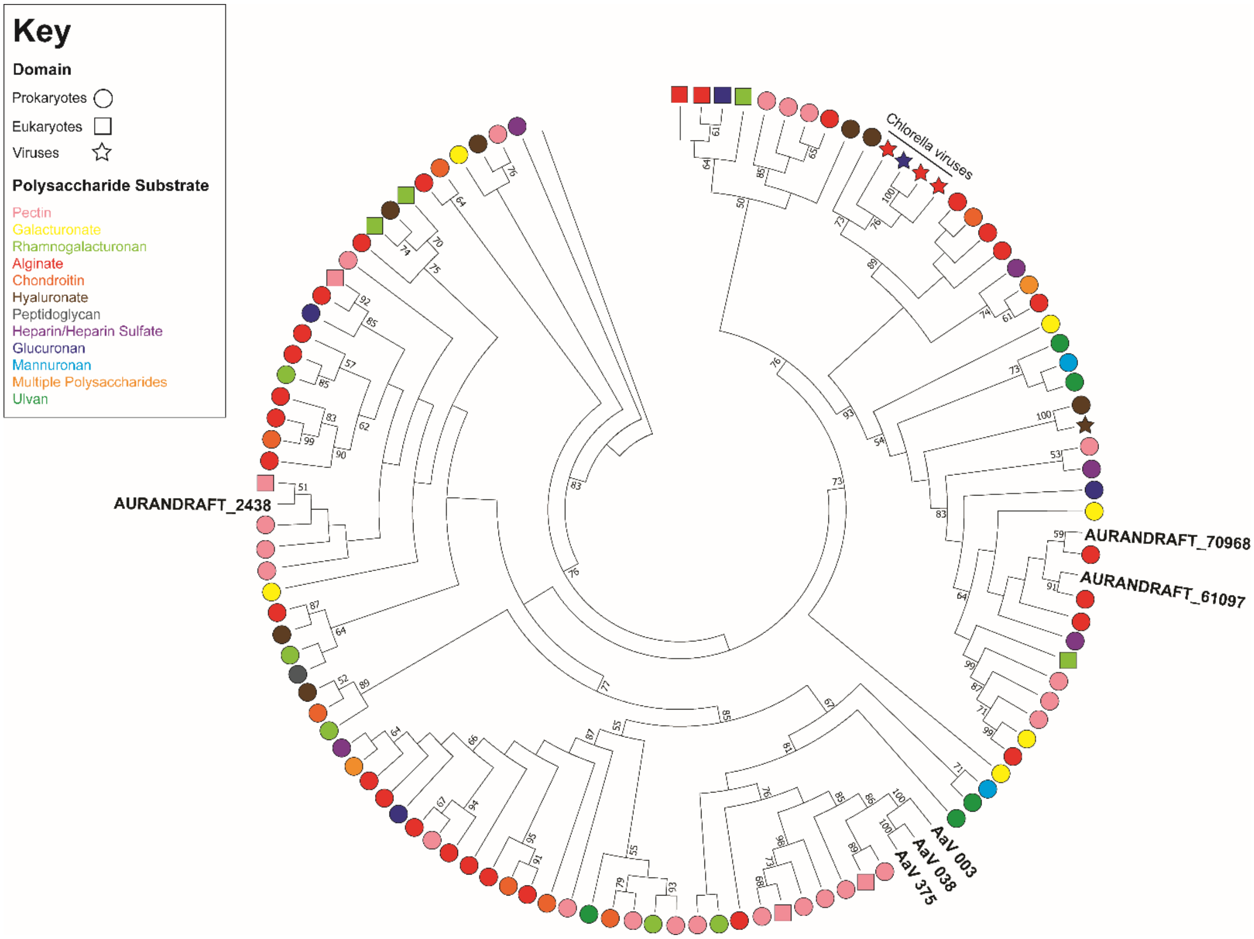

Supplemental Figure 2. Maximum likelihood phylogenetic of all characterized polysaccharide lyases within the Carbohydrate-Active Enzyme (CAZy) Database. Putative polysaccharide lyases from both *A. anophagefferens* and AaV genomes were also included.

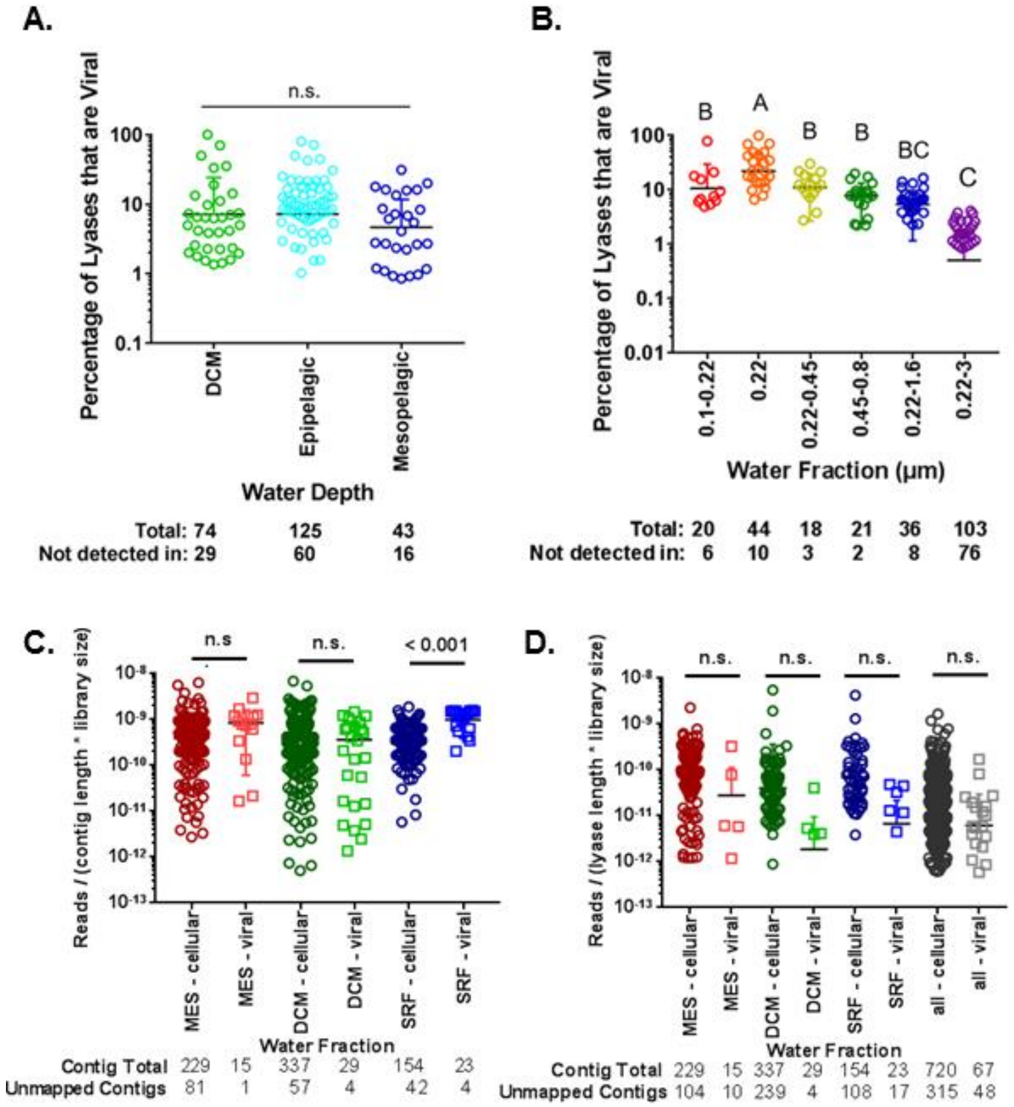

Supplemental Figure 3. Comparison of cellular and viral TARA Oceans-assembled contigs containing polysaccharides. A) Contigs separated by water depth at which samples were collected. One-way ANOVA p-values for differences between water depth are in Table 6. B) Contigs separated by size fraction of sample collected. One-way ANOVA p-values for differences between water depth are in Supplemental Data 1.2. C) Normalized Metagenomic reads mapped to the entire contigs. Reads from the metagenome the contig was assembled from were used to map back to each contig. D) Metatranscriptomic reads mapped to the polysaccharide lyases found on each contig. Reads from the metatranscriptomes at the same depth of the water column were used to map back to the polysaccharide lyases found on each contig. One-way ANOVA p-values for differences between cellular and viral mappings for metagenomic and metatranscriptomic analyses are in Supplemental Data 1.7 and 1.8, respectively.

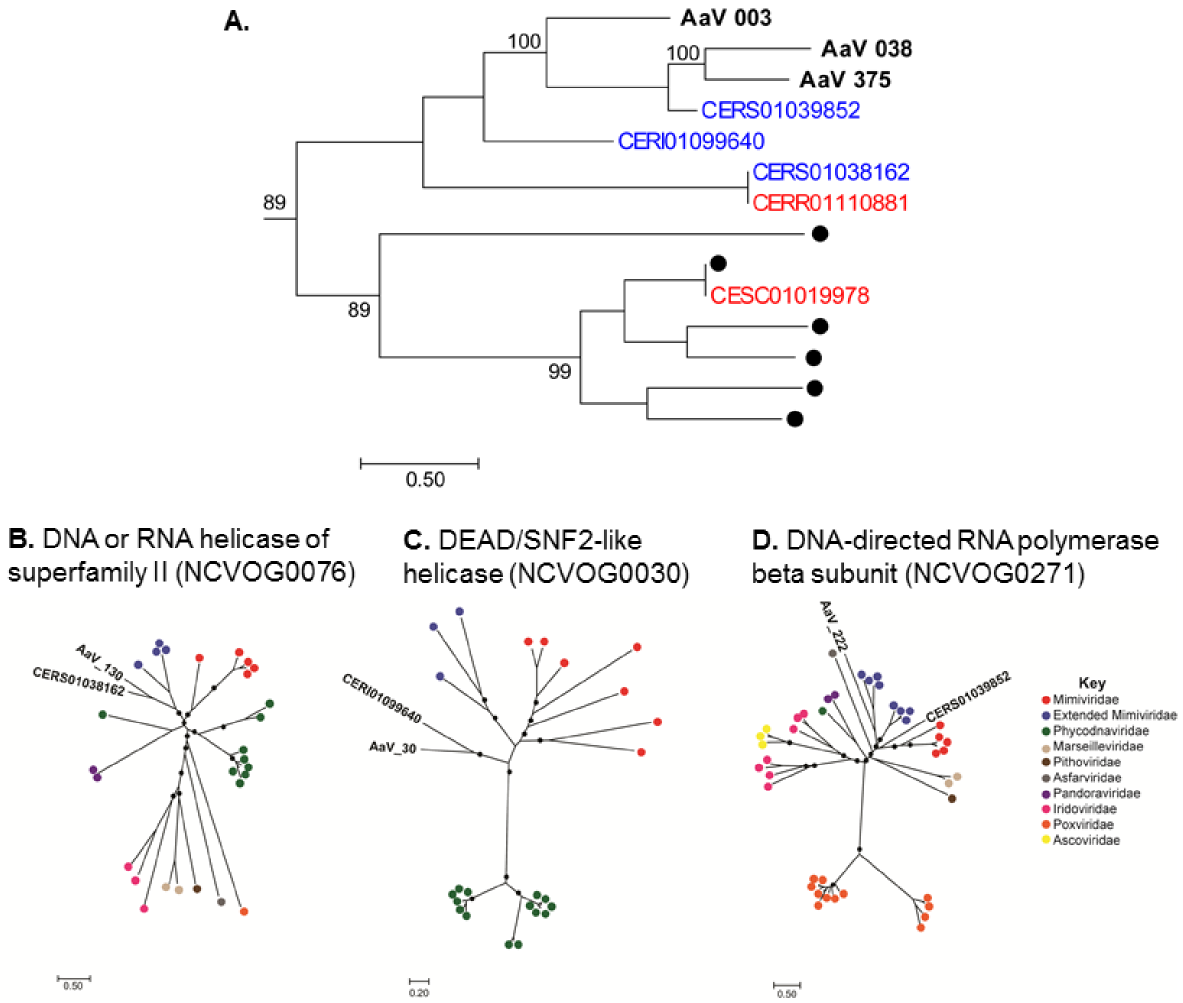

Supplemental Figure 4. Polysaccharide lyase containing viral contigs clustering with AaV. A) Subtree of maximum-likelihood phylogenetic placement of polysaccharide lyases from putative viral TARA Oceans metagenomic contigs containing the AaV pectate lyases. Contigs containing an NCVOG that the AaV genome possesses are in blue, while those with an NCVOG AaV does not possess are in red. Reference polysaccharide lyases from the Carbohydrate-Active Enzymes (CAZy) Database are denoted by black dots. B-D). Maximum likelihood phylogenetic trees of NCVOG found on TARA Oceans metagenomic contigs within AaV subtree of the maximum-likelihood phylogenetic placement of polysaccharide lyases from putative viral contigs. Viral families are denoted by colored dots. Black dots on branches represent bootstrap values > 0.5.

Supplemental Table 1. Mass spectrometry intensities and normalized intensities to MCP for proteins per particle for all the viral and host proteins detected within the proteomes. Coefficient of variation was determined for proteins detected in more than 1 proteomic run.

**Viral Proteins Detected**									
Accession Number	Gene Name	Average Sample 1	Average Sample 2	Average Sample 3	Proteins particle^{-1} Sample 1	Proteins particle^{-1} Sample 2	Proteins particle^{-1} Sample 3	average proteins particle -1	Coefficient of Variation
AaV_096	Capsid Protein	6.74E+08	8.15E+08	1.05E+09	5040.00	5040.00	5040.00	5040.00	0.00
AaV_024	Concanavalin A-like lectin/glucanase superfamily	1.24E+08	1.83E+08	3.19E+08	929.47	1135.22	1526.49	1197.06	20.69
AaV_276	Concanavalin A-like lectin/glucanase superfamily	9.52E+07	Cannot quant.	Cannot quant.	712.05			712.05	
AaV_386	Concanavalin A-like lectin/glucanase superfamily	1.41E+08	9.09E+07	1.06E+08	1057.35	562.36	507.56	709.09	34.87
AaV_080	Hypothetical	8.14E+07	1.07E+08	7.00E+07	609.22	663.80	334.83	535.95	26.86
AaV_314	Concanavalin A-like lectin/glucanase superfamily	7.39E+07	1.72E+07	1.36E+07	552.99	106.29	65.23	241.50	91.46
AaV_091	Hypothetical		3.65E+07	4.45E+07		226.11	212.80	219.45	3.03
AaV_214	Hypothetical	6.79E+07	1.88E+06	2.74E+05	508.18	11.63	1.31	173.71	136.17
AaV_038	pectate lyase	1.53E+07	3.45E+07	2.65E+07	114.35	213.51	126.98	151.61	29.07
AaV_074	Hypothetical	1.97E+07			147.67			147.67	
AaV_052	Hypothetical	3.20E+07	9.73E+06	4.37E+06	239.35	60.23	20.91	106.83	88.99
AaV_225	Hypothetical		1.58E+07	1.12E+07		97.50	53.81	75.65	28.88
AaV_231	Hypothetical	9.26E+06	2.10E+07	9.25E+05	69.30	130.12	4.43	67.95	75.53

AaV_232	Hypothetical	8.96E+06	Cannot quant.	Cannot quant.	67.03			67.03		
AaV_328	Hypothetical	7.46E+06			55.81			55.81		
AaV_175	Hypothetical	1.37E+07	4.77E+06	4.59E+06	102.45	29.53	21.98	51.32	70.71	
AaV_260	Hypothetical	6.78E+06			50.70			50.70		
AaV_306	putative ABC transporter family protein	4.64E+06			34.76			34.76		
AaV_103	Hypothetical	4.22E+06			31.60			31.60		
AaV_182	Hypothetical	3.70E+06			27.71			27.71		
AaV_250	Hypothetical	3.59E+06			26.83			26.83		
AaV_166	Hypothetical	3.20E+06			23.93			23.93		
AaV_281	putative membrane protein	3.02E+06			22.61			22.61		
AaV_242	putative DNA directed RNA polymerase II largest subunit	3.01E+06			22.51			22.51		
AaV_144	Hypothetical	2.90E+06			21.66			21.66		
AaV_062	Hypothetical	1.03E+06	5.00E+06	5.03E+06	7.68	30.97	24.05	20.90	46.72	
AaV_081	Hypothetical	2.51E+06			18.80			18.80		
AaV_211	putative mRNA capping enzyme	2.48E+06			18.53			18.53		
AaV_107	Hypothetical	2.41E+06			18.01			18.01		
AaV_136	Hypothetical	3.45E+06	2.49E+06	9.69E+05	25.84	15.42	4.63	15.30	56.60	
AaV_180	putative VV D6R-type helicase	2.02E+06			15.11			15.11		
AaV_158	metal dependent hydrolase		3.95E+06	3.86E+05		24.43	1.85	13.14	85.95	
AaV_329	Hypothetical	1.45E+06			10.87			10.87		

AaV_222	putative RNA polymerase beta subunit	1.32E+06			9.91			9.91	
AaV_286	Hypothetical	1.25E+06			9.34			9.34	
AaV_274	putative beta-1,4 galactosyltranferase	1.20E+06			8.97			8.97	
AaV_304	Hypothetical	1.05E+06			7.84			7.84	
AaV_279	Hypothetical	1.05E+06			7.84			7.84	
AaV_382	Hypothetical	9.58E+05			7.17			7.17	
AaV_247	putative capsid protein 2	9.56E+05			7.15			7.15	
AaV_269	putative ATP-dependent RNA helicase	8.36E+05			6.26			6.26	
AaV_174	DNA directed RNA polymerase K subunit	8.32E+05			6.22			6.22	
AaV_078	putative unsaturated glucuronyl hydrolase		Cannot quant.	Cannot quant.					
**Host Proteins Detected**									
Accession Number	Description	Average Sample 1	Average Sample 2	Average Sample 3	Proteins particle^{-1} Sample 1	Proteins particle^{-1} Sample 2	Proteins particle^{-1} Sample 3	average proteins particle -1	Coefficient of Variation
XP_009042681.1	hypothetical	2.80E+07	5.19E+08	2.89E+08	209.44	3212.54	1384.98	1602.32	77.11
XP_009032377.1	hypothetical		2.97E+06	3.11E+08		18.37	1488.59	753.48	97.56
XP_009042766.1	hypothetical	3.55E+07	6.03E+07	1.82E+08	265.27	373.27	868.84	502.46	52.30
XP_009037968.1	haemagglutinin	2.80E+07	1.38E+08	2.91E+07	209.44	855.03	139.27	401.25	80.29
XP_009038429.1	hypothetical	9.05E+07	4.66E+07	4.91E+07	677.38	288.38	234.77	400.18	49.29
XP_009034946.1	hypothetical		4.35E+07	1.01E+08		269.34	484.01	376.68	28.49
XP_009042897.1	hypothetical	3.41E+07	4.94E+07	1.18E+08	255.49	305.57	563.50	374.85	36.00
XP_009037560.1	hypothetical		3.55E+07	5.06E+07		219.67	241.89	230.78	4.82
XP_009035447.1	ADP-ribosylation factor	2.88E+07			215.63			215.63	
XP_009042844.1	hypothetical	1.90E+07	1.22E+07	6.69E+07	142.21	75.51	320.06	179.26	57.58

XP_009037423.1	alginate regulatory protein AlgP	6.58E+07	1.61E+06	3.68E+05	492.74	9.97	1.76	168.16	136.50
XP_009033628.1	hypothetical		3.28E+07	1.80E+07		202.93	86.23	144.58	40.36
XP_009043278.1	hypothetical	1.37E+07			102.52			102.52	
XP_009039958.1	hypothetical	2.10E+07	1.04E+07	6.84E+06	157.19	64.53	32.75	84.82	62.24
XP_009034221.1	hypothetical	8.59E+06			64.24			64.24	
XP_009042952.1	hypothetical	2.06E+07	1.32E+06	3.16E+06	154.25	8.18	15.13	59.19	113.67
XP_009038981.1	hypothetical	7.67E+06			57.41			57.41	
XP_009041980.1	hypothetical	7.58E+06			56.74			56.74	
XP_009037597.1	hypothetical		7.85E+06	1.33E+07		48.57	63.56	56.06	13.37
XP_009034199.1	Probable 26S proteasome non-ATPase regulatory subunit 3	7.27E+06			54.39			54.39	
XP_009036992.1	actinA	6.50E+06			48.63			48.63	
XP_009037302.1	actinA	6.50E+06			48.63			48.63	
XP_009038528.1	actinA	6.50E+06			48.63			48.63	
XP_009039473.1	actinA	6.50E+06			48.63			48.63	
XP_009043268.1	hypothetical	6.31E+06			47.24			47.24	
XP_009033043.1	hypothetical	5.07E+06			37.91			37.91	
XP_009043494.1	hypothetical	5.02E+06			37.56			37.56	
XP_009032842.1	UDP-galactose transporter		1.03E+07	1.77E+06		63.65	8.45	36.05	76.55
XP_009042736.1	K homology RNA-binding domain	3.98E+06			29.78			29.78	
XP_009038784.1	hypothetical		8.58E+06	5.48E+05		53.09	2.62	27.86	90.58
XP_009042729.1	superfamily; cl17037	3.48E+06			26.03			26.03	

XP_009034850.1	pH domain containing protein	3.23E+06			24.16			24.16	
XP_009039271.1	hypothetical		6.14E+06	4.88E+05		37.97	2.33	20.15	88.42
XP_009041703.1	hypothetical		6.14E+06	4.88E+05		37.97	2.33	20.15	88.42
XP_009033155.1	hypothetical		4.10E+06	2.77E+06		25.38	13.23	19.30	31.45
XP_009037422.1	putative hydroxylase	1.96E+06			14.69			14.69	
XP_009032689.1	histone	1.93E+06			14.42			14.42	
XP_009032767.1	histone	1.93E+06			14.42			14.42	
XP_009034195.1	histone	1.93E+06			14.42			14.42	
XP_009034430.1	histone	1.93E+06			14.42			14.42	
XP_009034585.1	histone	1.93E+06			14.42			14.42	
XP_009036131.1	histone	1.93E+06			14.42			14.42	
XP_009041154.1	histone	1.93E+06			14.42			14.42	
XP_009041608.1	histone	1.93E+06			14.42			14.42	
XP_009041779.1	histone	1.93E+06			14.42			14.42	
XP_009042081.1	histone	1.93E+06			14.42			14.42	
XP_009040236.1	hypothetical		1.32E+06	3.16E+06		8.18	15.13	11.66	29.79
XP_009033030.1	putative ribulose-1,5-bisphosphate carboxylase/oxygenase small subunit N-methyltransferase I	1.48E+06			11.11			11.11	
YP_003002020.1	photosystem I assembly protein Ycf4	1.38E+06			10.29			10.29	
XP_009033220.1	hypothetical	1.26E+06			9.47			9.47	
XP_009033273.1	heat shock protein 70	1.06E+06			7.92			7.92	

XP_009038582.1	Cell division protein FtsH	1.01E+06			7.59			7.59	
XP_009036072.1	hypothetical	9.42E+05			7.05			7.05	
XP_009037622.1	anaphase-promoting complex subunit 8-like protein	7.35E+05			5.50			5.50	
XP_009037653.1	putative urea active transport protein	5.99E+05			4.48			4.48	
XP_009034812.1	ARF1-directed GTPase-activating protein	3.13E+05			2.34			2.34	

Supplemental Table 2. Categories of reciprocal best BLAST hit pairs based on their NCVOG classification (Yutin et al., 2009).

Category	Number of RBH pairs	RBH pairs across families
Uncharacterized	215	34
DEAD/SNF2-like helicases	27	14
Transcription/RNA processing (RNA polymerases, Poxvirus early transcription factor, Ribonucease III, XrN 5-'3' exonuclease, mRNA capping enzyme)	39	9
MCP	21	13
Translation (translation initiation inhibitor yjgF family, eukaryotic translation intitation factor 4e)	7	4
Redox (Flavin-containing amine oxidoreductase, Erv1/Alr family oxidoreductase, Thioredoxin)	38	26
Kinases/Phosphatases (Serine/Threonine protein kinase, F10 like kinase, serine threonine phosphatase 2C, Dual specificity phosphatases (DSP); Ser/Thr and Tyr protein phosphatases, acid phosphatase class B)	22	7
Proteases (Ulp1 protease, trypsin-like serine protease, papain-like cysteine peptidase, metallopeptiase WLM, otubain-like protein)	10	2
Other (Patatin phospholipase, uracil-DNA glycosylase, histones, Zn-finger proteins, Poxvirus P4B major core protein, Esterase lipase superfamily, DNA polymerase X, collagen triple helix repeat containing protein, mannose-6P isomerase, glycosyltransferase, Ubiquitin, Nudix hydrolase, lipocalin family protein, FtsJ-like methyltransferase, AAA family ATPase	31	10
Total	410	119

Supplemental Table 3. Classification of putative viral contigs containing polysaccharide lyases into viral families by best BLAST hit to NCVOG on contig.

Family	Count	Family/Total Viral
Pithoviridae	4	0.008
Phycodnaviridae	183	0.367
Mimiviridae	142	0.285
Pandoraviridae	50	0.100
Iridoviridae	7	0.014
Ascoviridae	68	0.136
Poxviridae	15	0.030
Extended Mimiviridae	30	0.060
Total	499	1.000

Supplemental Table 4. List of sequencing data used in this study. [*] Assembled contigs downloaded from European Nucleotide Archive from https://www.ebi.ac.uk/ena/about/tara-oceans-assemblies. [†] Run accessions downloaded from European Nucleotide Archive.

TARA Station 122 Assembled Contigs[*]	CEOO01	CEPX01	CERG01	CESP01	CETY01	CEVH01	TARA Station 122 – Metagenomes[†]
CENF01	CEOP01	CEPY01	CERH01	CESQ01	CETZ01	CEVI01	ERR594284
CENG01	CEOQ01	CEPZ01	CERI01	CESR01	CEUA01	CEVJ01	ERR594301
CENH01	CEOR01	CEQA01	CERJ01	CESS01	CEUB01	CEVK01	ERR594304
CENI01	CEOS01	CEQB01	CERK01	CEST01	CEUC01	CEVL01	ERR594305
CENJ01	CEOT01	CEQC01	CERL01	CESU01	CEUD01	CEVM01	ERR598948
CENK01	CEOU01	CEQD01	CERM01	CESV01	CEUE01	CEVN01	ERR594292
CENL01	CEOV01	CEQE01	CERN01	CESW01	CEUF01	CEVO01	ERR594322
CENM01	CEOW01	CEQF01	CERO01	CESX01	CEUG01	CEVP01	ERR594307
CENN01	CEOX01	CEQG01	CERP01	CESY01	CEUH01	CEVQ01	ERR598999
CENO01	CEOY01	CEQH01	CERQ01	CESZ01	CEUI01	CEVR01	ERR594306
CENP01	CEOZ01	CEQI01	CERR01	CETA01	CEUJ01	CEVS01	ERR598992
CENQ01	CEPA01	CEQJ01	CERS01	CETB01	CEUK01	CEVT01	ERR594309
CENR01	CEPB01	CEQK01	CERT01	CETC01	CEUL01	CEVU01	
CENS01	CEPC01	CEQL01	CERU01	CETD01	CEUM01	CEVV01	TARA Station 122 – Metatranscriptomes[†]
CENT01	CEPD01	CEQM01	CERV01	CETE01	CEUN01	CEVW01	ERR3587201
CENU01	CEPE01	CEQN01	CERW01	CETF01	CEUO01	CEVX01	ERR3587197
CENV01	CEPF01	CEQO01	CERX01	CETG01	CEUP01	CEVY01	ERR3587126
CENW01	CEPG01	CEQP01	CERY01	CETH01	CEUQ01	CEVZ01	ERR3587160
CENX01	CEPH01	CEQQ01	CERZ01	CETI01	CEUR01	CEWA01	ERR3587125
CENY01	CEPI01	CEQR01	CESA01	CETJ01	CEUS01	CEWB01	ERR3587151
CENZ01	CEPJ01	CEQS01	CESB01	CETK01	CEUT01	CEWC01	ERR3587172
CEOA01	CEPK01	CEQT01	CESC01	CETL01	CEUU01	CEWE01	
CEOC01	CEPL01	CEQU01	CESD01	CETM01	CEUV01	CEWF01	
CEOD01	CEPM01	CEQV01	CESE01	CETN01	CEUW01	CEWG01	

CEOE01	CEPN01	CEQW01	CESF01	CETO01	CEUX01	CEWH01
CEOF01	CEPO01	CEQX01	CESG01	CETP01	CEUY01	CEWI01
CEOG01	CEPP01	CEQY01	CESH01	CETQ01	CEUZ01	CEWJ01
CEOH01	CEPQ01	CEQZ01	CESI01	CETR01	CEVA01	CEWK01
CEOI01	CEPR01	CERA01	CESJ01	CETS01	CEVB01	CEWO01
CEOJ01	CEPS01	CERB01	CESK01	CETT01	CEVC01	CEWP01
CEOK01	CEPT01	CERC01	CESL01	CETU01	CEVD01	CEWQ01
CEOL01	CEPU01	CERD01	CESM01	CETV01	CEVE01	CEWR01
CEOM01	CEPV01	CERE01	CESN01	CETW01	CEVF01	CXWF01
CEON01	CEPW01	CERF01	CESO01	CETX01	CEVG01	
